# Circumstances of Percutaneous Sharps Injuries in German Healthcare Workers—An Analysis of the Ten-Year Period from 2015 to 2024 Based on Accident Insurance Data

**DOI:** 10.3390/ijerph23040412

**Published:** 2026-03-25

**Authors:** Madeleine Dulon, Johanna Stranzinger, Dana Wendeler, Albert Nienhaus

**Affiliations:** Berufsgenossenschaft für Gesundheitsdienst und Wohlfahrtspflege, 22089 Hamburg, Germany; johanna.stranzinger@bgw-online.de (J.S.); albert.nienhaus@bgw-online.de (A.N.)

**Keywords:** sharps injuries, needlestick injuries, health personnel, safety-engineered device, occupational safety

## Abstract

**Highlights:**

**Public health relevance—How does this work relate to a public health issue?**

- Twenty years after the introduction of the European legislation for the prevention of sharp injuries and the national implementation in workplaces, annual reports of sharps injuries remain at almost unchanged levels.
- Around one third of sharps injuries involved a feature with an integrated safety mechanism.

**Public health significance—Why is this work of significance to public health?**

- Around one third of sharps injuries occurred after use and were related to disposal.
- Insulin injection devices were the most frequent cause of sharps injuries among healthcare workers in nursing homes.

**Public health implications—What are the key implications or messages for practitioners, policy makers and/or researchers in public health?**

- Training on the proper disposal of used sharp instruments should be provided to all occupational groups involved.
- Healthcare workers and trainees should be offered regular training to feel confident in the use of safety-engineered devices.

**Abstract:**

Despite the implementation of safety-engineered devices (SEDs) in Germany, percutaneous sharps injuries (PSIs) caused by medical devices remain a major occupational risk for healthcare workers. The aim of this study was to analyze the frequency of PSIs and the circumstances of SED-associated PSIs in hospitals, medical practices, and nursing homes. Routine data from a statutory accident insurance provider for 2015–2024 were used to analyze PSI trends (n = 481,575), and survey data from online questionnaires were used to analyze circumstances of PSIs (n = 791). Routine data showed a slight decline (6.1%) in PSIs over the past 10 years across all sectors. Hospitals and medical practices had the highest rates (30.2 and 21.6 PSIs per 1000 full-time equivalents, respectively). The devices most frequently involved were blood collection needles in hospitals and medical practices and insulin pens in nursing homes. Overall, 43.1% of PSIs were related to the improper disposal of used devices. Around 31.1% of PSIs were associated with SEDs. Around 33% of SED-related injuries occurred during disposal. High workload and distraction were the most frequently reported causes of injuries. Regular training should be provided to raise staff awareness of the proper handling and disposal of used devices.

## 1. Introduction

Percutaneous sharps injuries (PSIs) are a common occupational hazard among healthcare workers (HCW) and increase the risk of blood-borne infections such as hepatitis B virus, hepatitis C virus, and human immunodeficiency virus (HIV). Between 2000 and 2020, the pooled global one-year prevalence of PSIs among HCW was 39.0–44.5% while the lifetime prevalence was 56.6% [1,2]. Globally, the one-year prevalence of PSIs among HCW varied substantially and ranged from 7.7% to 43.2% [3]. For Europe, the estimated pooled one-year prevalence of PSIs among HCW was 31.8% with values ranging between 18.9% and 47.3% [3]. Compared with the frequency of PSIs, the transmission of pathogens such as HIV or hepatitis viruses following occupational exposure to blood has rarely been demonstrated in epidemiological studies and only after deep injuries involving hollow instruments used in venous or arterial procedures [4].

A key strategy for reducing the risk of injuries among HCW is the use of safety-engineered devices (SEDs). Following the replacement of conventional needles with SEDs, the effectiveness of various interventions to prevent PSIs has been assessed. Findings regarding the accident dynamics have been conflicting and have ranged from low-quality and inconsistent evidence that SEDs prevent PSIs to moderate-quality evidence that their use in intravenous injections and phlebotomy procedures reduces PSI rates [5,6]. Studies examining PSI trends over periods of 10 to 20 years reported that the number of PSIs among care staff decreased following the implementation of SEDs [7,8]. A meta-analysis including studies published between 1993 and 2016 showed that countries that adopted needlestick safety and prevention regulations mandating the use of SEDs had lower PSI incidence among HCW than countries without comparable policies [9].

With the European Directive 2010/32, essential requirements for the prevention of PSIs in the hospital and healthcare sectors have been established. These requirements were transposed into national law by the Member States [10]. In Germany, this took place as part of the revision of the Biological Agents Ordinance in 2013. Within its scope of application, the “Technical rule for biological agents in healthcare and welfare facilities” (TRBA 250) specifies the requirements of the Biological Agents Ordinance. Since March 2014, healthcare institutions have been required to implement measures to protect employees from injuries caused by sharp and pointed medical instruments. A central protective measure is the use of instruments with an integrated safety mechanism whenever transmission of infectious quantities of blood may occur [11]. Further protective measures include the disposal of sharp medical instruments exclusively in designated containers, the prohibition of reinserting used needles into the protective cap (recapping), and the requirement for standardized and anonymized documentation of PSIs [11].

It is estimated that there are around 1.2 million PSIs in European healthcare facilities each year [12]. A survey commissioned by the European Biosafety Network (EBN) in spring 2021 found an average increase in PSIs of 23% ranging from +9% in Italy to +32% in Germany [13,14]. The occupational physicians surveyed from 80 hospitals, each with more than 500 beds, cited various situations related to the COVID-19 pandemic as reasons for the increase in PSIs. These included the implementation of vaccination programs at the beginning of 2021, the increase in workplace stress, and shortages of SEDs and personal protective equipment.

In Germany, employers are required to report occupational accidents that result in incapacity for work lasting more than three days or in death to the statutory accident insurance provider within three days. The accident report is used to record circumstances necessary for initiating the determination procedure and for tasks related to prevention and rehabilitation. These also serve as the basis for documenting characteristics used in occupational accident statistics. Most accident reports relating to PSIs are not associated with long-term incapacity for work. Instead, they are submitted to the statutory accident insurance provider by the treating doctor together with the invoice for treatment or post-exposure prophylaxis.

One of the German accident insurance providers is the accident insurance provider BGW, a compensation board which is tasked with preventing work-related accidents and occupational diseases. Those insured by the BGW are employed in non-governmental facilities in the health and welfare sectors. The BGW supports its member companies by conducting risk assessments following injuries caused by medical devices and provides a freely accessible online tool on its website for PSI-related occupational accidents [15]. This tool was developed to support member companies in analyzing and documenting occupational accidents involving contact with blood and bodily fluids and establish a computerized database of PSI records in order to monitor the effects of national needlestick safety and prevention regulations. To obtain these data, an online questionnaire was made available via a link to HCWs who had reported an accident involving a PSI using the online tool on the BGW website.

The aims of the study were to describe the frequency of PSIs over the past 10 years based on routine data from accident insurance records and to analyze the circumstances and causes of needlestick injuries based on survey data.

## 2. Materials and Methods

### 2.1. Routine Data

The analysis is based on accident reports from the BGW on PSIs between 2015 and 2024. Four variables were selected from the accident statistics to identify PSIs: type of injury, injured body part, causative object, and case-specific information. Included were cases involving superficial injuries to the hands and fingers, as well as injuries caused by infectious material involving a sharp or cutting medical instrument such as a needle, syringe, or scalpel, provided the case was marked with the scenario ‘post-exposure prophylaxis’. This scenario was interpreted as indicating exposure to blood from a patient with suspected or confirmed infection with HIV or hepatitis viruses. The sample consisted of 481,575 data records on reportable and non-reportable PSIs.

### 2.2. Survey Data

The survey analysis was based on data collected via the online tool on the BGW website from insured persons who had sustained a needlestick and sharps injury (NSSI). An NSSI was defined as an injury involving a contaminated sharp medical device, irrespective of whether or not the wound was bleeding. Data were collected between February 2022 and March 2025. A total of 8355 insured persons received an invitation letter, including study information, within one month of their accident report being recorded by the BGW. By the data collection deadline of 31 March, 1067 completed questionnaires had been received on the BGW online platform. Of these, 276 participants were excluded (25.9%) based on the following exclusion criteria: (1) not working in the selected settings (n = 33); (2) using devices not contaminated with blood (n = 150); (3) without information on whether the device was equipped with a safety mechanism (n = 93). In total, 791 participants satisfied the inclusion criteria. Fewer than 1% of those contacted in writing (476 of 8355) were included in the final sample.

Participants were characterized by sex, age, occupational role, healthcare sector, and whether an SED was involved in the NSSI. In order to describe the circumstances of the accident, information on the device involved and the activity being performed at the time of injury was assessed. Information on the most relevant reason for the accident was collected, and answers were grouped into the following categories: high workload, lack of attention, unexpected patient movement, distraction by surrounding activities, problems related to the sharps container, and problems related to the safety device. For questions about safety devices, explanations of passive and active safety mechanisms were added as explanatory texts and pop-up windows. Data are presented separately for three healthcare settings: (1) hospitals, including clinics, rehabilitation clinics, and dialysis centers; (2) medical practices, including any specialty, dentistry, and laboratories; and (3) nursing homes, including elderly care facilities, hospices, and outpatient care services.

Data were analyzed descriptively using SPSS Version 28 (IBM Corp., Armonk, NY, USA) and presented as absolute and relative frequencies. To assess the frequency of PSI across different sectors, we used full-time equivalents (FTE) to calculate PSI rates per 1000 FTE. One FTE corresponds to one full-time position or two part-time positions. To describe the extent to which safety features were used, a percentage was calculated for the number of NSSIs associated with SEDs separately for each category of the respective characteristics.

## 3. Results

### 3.1. Routine Data

**Figure 1:**
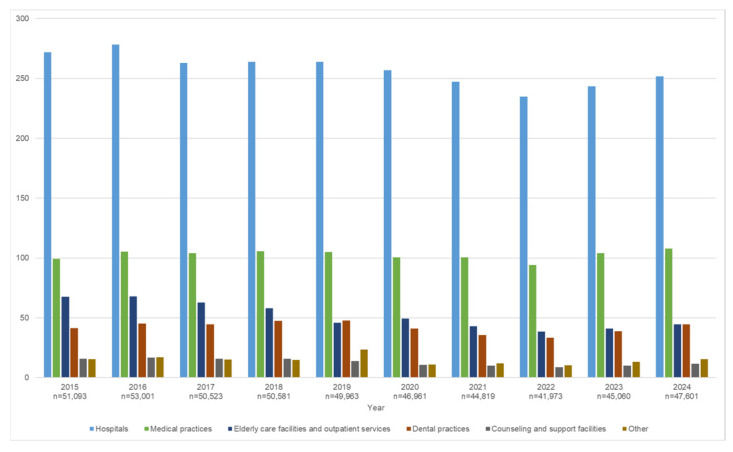
Percutaneous sharps injuries (per 100) separated by sector for the years 2015 to 2024, registered by the accident insurance provider BGW.

Based on accident statistics, between 42,000 and 53,000 PSIs were recorded each year by the BGW between 2015 and 2024 (Figure 1). The number of PSIs reported each year was on average 51,032 up to 2019 and on average 45,283 thereafter. The only exception was 2020, when they fell to just under 42,000. Overall, annual numbers of reported PSIs remained almost unchanged with a slight decline (6.1%) over the 10-year period. In 2022, reporting numbers were, on average, around 16% lower than in 2019. The pre-pandemic levels were reached again in 2024. This pattern of a decreasing number of reports up to 2022 followed by an increase up to 2024 was observed across all sectors. The trend was more pronounced in two sectors (counseling and support facilities and dental practices) with decreases and subsequent increases of more than 30% in each case) and less pronounced in hospitals and medical practices (with decreases and increases of around 10%). Reportable PSI made up less than 1% of PSI reports in all years and also decreased between 2019 and 2022.

In all years, the highest proportion of reported PSI cases occurred in hospitals with considerably lower proportions in the other sectors (Figure 1). In 2024, around 50% of PSI reports came from hospitals, 25% from medical practices, and around 9% each from elderly care facilities and dental practices (Table 1). Nursing staff were most frequently involved in PSIs, followed by physicians and medical assistants. These three occupational groups accounted for almost 80% of all PSIs. Around 80% of the registered PSIs concerned women. Among medical and dental assistants as well as nursing staff, 90% of PSIs concerned women.

The number of reported PSIs per 1000 FTE varied between 30.2 (in hospitals) and 1.4 cases (in counseling and support facilities) (Table 2). The number of PSI reports per 1000 FTE was around 3.5-fold higher in hospitals than the average across all BGW sectors (8.8 per 1000 FTE) and around two-fold higher in medical and dental practices.

### 3.2. Survey Data

Descriptive statistics of the study population are shown in Table 3. Almost 50% of the participants worked in medical practices (376 of 791), 41.3% in hospitals (327 of 791, and 11.1% in care facilities (88 of 791). In medical practices and care facilities, women accounted for almost 90% of NSSI cases; in hospitals, the proportion was lower. The median age was 36 years in hospitals and 33–34 years in the two other settings.

Almost all reports of NSSIs from care facilities came from nursing staff, while in medical practices, more than 50% were submitted by medical assistants. In hospitals, around 50% of reports originated from nursing staff and around 33% from medical staff. In all three settings, trainees were involved in around 12.5% of NSSIs. Staff in cleaning services accounted for only a few cases. More than 80% of employees of hospitals and medical practices sought medical advice or treatment compared with just over 50% of employees from care facilities. Only a small number of NSSIs resulted in incapacity for work lasting more than three days. In 31.1% of NSSIs (246 of 791), an SED was used when the injury occurred. This proportion was higher in hospitals and lower in care facilities. In 45.9% of cases (113 of 246), the SEDs were equipped with a passively activated safety mechanism.

22.3% of participants from hospitals and 13.3% of participants from medical practices reported having worked 7 h or more at the time of the NSSI. Needles for intravenous procedures such as blood sampling were frequently involved in NSSIs in hospitals and medical practices as well as in SED-associated NSSIs (Table 4). In care facilities, insulin pen needles were involved in around 50% of NSSIs, while SEDs were involved in only a small number of cases. The most frequent procedures involved in NSSI in hospitals were suturing and surgery caused by surgical suture needles and scalpels. In medical practices and care facilities, NSSIs most frequently occurred during disposal and setting-up; 43.1% of NSSIs occurred during cleaning, tidying up, recapping and disposal (i.e., after the actual use of the instrument) (341 of 791). Even when an SED was used, around 33% of NSSIs occurred in connection with disposal, recapping, and cleaning activities (80 of 246).

In hospitals and medical practices, the most common reasons for NSSIs were high workload and time pressure as well as distraction caused by activities in the surrounding. Unexpected patient movements were the most common reason for NSSIs in care facilities and the third most common reason in hospitals and medical practices (Table 4). Problems with sharps containers and with the handling of SEDs were also cited as reasons for NSSIs. Around 25% of NSSIs during the use of an SED (n = 246) were attributed to a product failure and 14% to a lack of experience in using the device.

## 4. Discussion

Using routine data, we described numbers of registered PSIs over the past 10 years. We used survey data to identify circumstances of NSSIs in specific healthcare settings.

### 4.1. Routine Data

The analysis of accident statistics for PSI frequencies over the past 10 years was prompted by the European EBN study, which reported a 32% increase in PSIs in Germany over the first two years of the COVID-19 pandemic (2020/2021) [13]. This trend cannot be observed on the basis of BGW reporting data; instead, PSI reports declined across all healthcare sectors after 2016 and reached a low point in 2022, followed by an increase to pre-pandemic levels up to 2024 (Figure 1). According to the EBN study, the increase was especially pronounced in blood testing and emergency care settings in Germany. These two activities cannot be analyzed using routine data. Hospitals and medical practices were analyzed as a proxy; PSI numbers also declined in these settings between 2019 and 2022.

To show the development of PSI case numbers across all sectors of the health service in Germany, the data of the German Social Accident Insurance, the umbrella organization of German accident insurance institutions (DGUV) is suitable, as these statistics also include PSI cases from hospitals under public ownership (e.g., university clinics). For reported PSI cases, the number decreased by 50% between 2015 and 2019 with a low point in 2020 (a further decrease of 26%); in 2021, there was an increase of almost 63% compared with 2020, followed by a levelling off at the pre-pandemic level [16]. The development is comparable to that shown in the statistics of the BGW, with the difference that the data of the DGUV is based exclusively on PSI cases of incapacity for work lasting longer than 3 days.

In contrast to the BGW data, a Chinese study reported a 4.3-fold increase in the risk of PSIs in 2021 compared with 2019 [17], and a Turkish study found a significant increase in PSI rates among care staff between 2019 and 2020 [18]. A Croatian study observed a decrease in the number of PSIs during the first two years of the COVID-19 pandemic (2020–2022) [19]. However, after adjusting for factors such as patient numbers and hospital activities, the findings showed that PSI rates were significantly higher during the first two years of the pandemic (2020 and 2021) than in the two years prior (2018 and 2019) [19]. Although some studies were aimed at evaluating the possible effects of the pandemic on the number of PSIs, the results were inconclusive, and the study designs did not allow for more detailed analysis [18,20]. In assessing these opposing trends, it should be taken into account that the BGW data are based on registered data, whereas the other data are derived from study surveys. It is assumed that there is a high non-response rate for PSI reports [21]; this could explain the differences.

### 4.2. Survey Data

Our investigation into the circumstances of NSSIs should be assessed against the backdrop of the Needlestick Safety and Prevention Act—which entered into force in the United States around 20 years ago—and Directive 2010/32/EU on the prevention of sharps injuries in the hospital and healthcare sector [10,22]. For the present analysis, the sample consisted of just under 800 participants. Although the sample represents only a small fraction of the NSSIs reported annually to the BGW, it nevertheless allows (1) analysis of NSSIs in the three areas in which a large proportion of BGW-insured persons are employed and (2) comparison with an earlier study based on the same data source. As in the survey conducted five years earlier, hollow-bore needles were most frequently involved in NSSIs across all three settings [23] and—as reported by other authors—intravenous and subcutaneous needles and the associated activities were most commonly involved [24]. Other authors have also pointed out the high proportion of insulin pen needles involved in NSSIs in elderly care facilities [25,26].

Healthcare workers in a range of settings are at considerable risk of suffering an NSSI when disposing of sharp instruments [27]. Many of the problems described in connection with sharps containers, tidying-up activities, or recapping occurred only after the actual use of the instrument [24]. Recapping is regarded as one of the most common causes of NSSIs internationally but was around six-fold lower in our study and accounted for only 5% of cases (42 of 791) [27]. Under TRBA 250, reinserting used needles into the protective cap is no longer permitted in Germany unless a procedure that allows the needle to be safely recapped with one hand is used (e.g., when using a cap holder for local anaesthesia in dentistry) [11]. As described by other authors, high workload, long working hours, distraction, and unexpected patient movement were the main causes of NSSIs in all three settings [8,20,24,27].

### 4.3. Safety-Engineered Devices and NSSI

In this study, 31.1% of the NSSIs were associated with an SED. In other European studies, this was around 20% [24,28]. In a 2019 study with a sample drawn from the same data source, the proportion of SED-associated NSSIs was also around 30% [23]. In the current survey, only medical practices reported a rate of SED-associated NSSIs that was 17 percentage points lower. The data do not allow conclusions as to whether this difference was due to greater staff experience in handling SEDs or to the lower availability of SEDs in the practices concerned.

Other studies have also reported that the proportion of needles equipped with a safety mechanism varies depending on the type of needle used (approximately 50% or 90% when subcutaneous or blood collection needles were used) [20]. In our data, the corresponding proportions of devices equipped with a safety mechanism were only half as high in both cases. The EU Directive does not explicitly recommend the use of SEDs for subcutaneous needles; by contrast, the WHO advocates the use of SEDs for subcutaneous injections and justifies this with the large number of subcutaneous injections performed worldwide [1,21]. Assessing the risk of infection following an NSSI involving a subcutaneous needle is difficult because valid data on the risk of transmission are lacking [29] and so far only cases involving deep injuries and venous or arterial procedures have been described [4]. In Germany, statutory health insurance has covered safety pen needles and safety lancets since 2019 [30]. In elderly care facilities, this may contribute to preventing NSSIs during insulin administration. However, studies show that needlestick injuries cannot be completely ruled out when SEDs are used but are far less frequent [5,31].

The importance of training in the handling of SEDs is underscored by the large number of injuries that occurred during the disposal of an SED or indicated problems with activating the safety mechanism. Observations from a study conducted in the second year of the pandemic further underline the need for regular training in the handling of SEDs. Compared with a survey conducted in 2017, the level of participation in training courses had declined—as had knowledge of safe working practices with needles and their safe disposal; this was attributed to enforced social distancing in the first year of the pandemic [20]. Studies have shown that younger nursing staff, particularly those with little professional experience, should not be overlooked when providing instruction on safe working practices with SEDs [8,31]. Various authors emphasize the importance of combining the introduction of SEDs with regular training sessions and report that this combination could reduce the risk of NSSIs [32,33].

### 4.4. Limitations

Work-related accident statistics consist of claims data collected during insurance administration processes. Even though routine data are generally assumed to be free from selection bias [34], there are some limitations. The first limitation relates to the true prevalence of PSIs. It is likely that PSIs are under-reported because of the high proportion of unreported cases, particularly among physicians [21]. Another limitation is that the routine data do not allow to assess the risk of PSIs depending on gender because the total number of women working in the respective sector or in a given profession is not known.

Although the survey data are based on only a small sample, they included occupational groups that are rarely represented in study populations like non-hospital settings but are highly relevant from a prevention perspective. In fact, the analysis for the sector nursing homes is based on a very small sample, so the results for this sector are of limited relevance. It should be noted that the survey data concerning the circumstances of the NSSIs consists on self-reported data. This information bias applies particularly to whether the relevant device involved in the NSSI was an SED or a non-SED. There are indications that there were problems in classifying devices as SEDs in all three groups. In the case that a needle was misclassified as an SED, this would lead to overestimation of SED-associated NSSIs.

## 5. Conclusions

Despite national regulations and the implementation of precautionary measures, including needles with safety features, PSIs remain a considerable risk across all occupational groups and settings in the German healthcare system. The PSI data of the BGW have shown a slight downward trend over the last 10 years. It cannot be determined whether this positive development is attributable to the use of safety devices. Our findings show that the proportion of injuries associated with an SED remained unchanged at around 30% in recent years. The improper disposal of used instruments was a risk factor in all three sectors. Training programs with regular refresher sessions for all occupational groups affected by PSIs are required in order to raise awareness of the risk of injury during the disposal of sharp instruments.

## Figures and Tables

**Table 1:** Percutaneous sharps injuries ^1^ by sector and profession in 2024 (N = 47,601), registered by the accident insurance provider BGW.

Variable	N	%	% Female
Healthcare sector			
Hospitals	25,193	52.9	69.4
Medical practices	10,795	22.7	89.8
Elderly care facilities and outpatient services	4467	9.4	81.3
Dental practices	4447	9.3	91.6
Counseling and support facilities ^2^	1149	2.4	76.7
Other ^3^	1550	3.3	78.6
Profession			
Nurse, geriatric nurse	17,300	36.4	81.2
Physician	9871	20.7	50.4
Medical assistant	9657	20.3	93.6
Dental assistant and hygienist	3576	7.5	95.9
Nursing assistant, trainee nurse	1657	3.5	78.5
Dentist	782	1.6	71.7
Cleaning staff	370	0.8	87.8
Other professions ^4^	4388	9.2	75.7

^1^ Reportable and not reportable cases. ^2^ Children’s and youth welfare facilities, counseling services. ^3^ Administration, education, therapeutic practices, pharmacy, vocational rehabilitation, assisted workplaces for people with disabilities, veterinary medicine, childcare, hairdressing, beauty and wellness, pest control. ^4^ Veterinarians, veterinary assistants, employees in assisted workplaces for people with disabilities, medical and dental prosthetic technicians.

**Table 2:** Number of percutaneous sharps injury (PSIs) and number of reported cases per full-time equivalents (FTE) in 2024 by sector; data from the accident insurance provider BGW.

Sector ^1^	Full-Time Equivalents	PSI Reports	
		N	Per 1000 FTE
Hospitals	833,999.4	25,193	30.2
Medical practices	499,375.4	10,795	21.6
Dental practices	237,125.2	4447	18.8
Elderly care facilities and outpatient services	1,077,006.3	4467	4.1
Counseling and support facilities	836,378.9	1149	1.4
Other sectors	1,933,527.4	1550	0.8
All BGW sectors	5,417,412.6	47,601	8.8

^1^ Composition as described in Table 1.

**Table 3:** Characteristics of healthcare personnel with needlestick and sharps injuries (NSSIs) by healthcare setting; survey data between 2022 and 2024 (n = 791).

Variable	Hospital ^1^ (N = 327)		Medical Practice ^2^ (N = 376)		Nursing Home ^3^ (N = 88)	
	n	%	n	%	n	%
Sex female	248	75.8	342	91.0	77	87.5
Age (years) (median with 25th + 75th Percentiles)	36 (27/48)		34 (23/47)		33 (25/42)	
Profession						
Nurse, geriatric nurse	125	38.2	22	5.9	59	67.0
Nurse assistant	5	1.5	1	0.3	12	13.6
Physician, dental physician	103	31.5	69	18.4	1	1.1
Doctor’s assistant ^4^	44	13.5	218	58.0	1	1.1
Nurse trainee	41	12.5	59	15.7	11	12.5
Other profession ^5^	9	2.8	7	1.9	4	4.5
Time between start work and NSSI (hours)						
Not known, ≤6	254	77.7	326	86.7	80	90.9
>6	73	22.3	50	13.3	8	9.1
Medical consultation and treatment after NSSI	274	83.8	289	76.9	50	56.8
-of which on sick leave for more than three days	4/274		8/289		3/50	
Use of a safety engineered device	120	36.7	106	28.2	20	22.7
Type of safety mechanism						
Active	58	48.3	59	55.7	6	30.0
Passive	58	48.3	44	41.5	11	55.0
Unknown	4	3.4	3	2.8	3	15.0

^1^ Including hospitals, rehabilitation clinics, and dialysis wards. ^2^ Including all specialist facilities, dentistry, and laboratories. ^3^ Including elderly care facilities, outpatient and hospice care, and others (e.g., facilities for the disabled). ^4^ Including medical assistants, dental assistants, and oral hygienists. ^5^ Including staff from emergency services, pharmacies, cleaning, and kitchen.

**Table 4:** Number of devices, activities and reasons associated with needlestick and sharps injuries (NSSIs) and percentage of NSSI associated with a safety feature (SED) separated by healthcare setting ^1^; survey data between 2022 and 2024 sorted by frequency in hospitals (n = 791).

Variable	Hospital ^1^		Medical Practice ^2^		Nursing Home ^3^	
	Total	Associated with an SED	Total	Associated with an SED	Total	Associated with an SED
	n = 327	n = 120	n = 376	n = 106	n = 88	n = 20
	n	n (% of Total)	n	n (% of Total)	n	n (% of Total)
Device						
Intravenous needle ^2^	119	70 (58.8)	149	79 (53.0)	14	4 (28.6)
Suture needle, surgical device	74	4 (5.4)	19	2 (10.5)	0	0 (0.0)
Subcutaneous needle	70	31 (44.3)	148	19 (12.8)	21	6 (28.5)
Insulin pen needle	26	8 (30.8)	0	0 (0.0)	46	8 (17.4)
Scalpel	25	6 (24.0)	21	2 (9.5)	1	0 (<0.1)
Other device ^3^	13	1 (7.7)	39	4 (10.3)	6	2 (33.3)
Activity when injury occurred						
Suturing/surgery	76	12 (15.8)	24	3 (12.5)	0	0 (0.0)
Blood sampling	69	49 (71.0)	85	58 (68.2)	4	2 (50.0)
Disposal and setting up of contaminated devices ^4^	64	26 (40.6)	113	27 (23.9)	34	5 (14.7)
Administering an injection	41	16 (39.0)	52	8 (15.4)	23	9 (39.0)
Tidying and cleaning up	30	12 (40.0)	49	2 (4.1)	9	1 (11.1)
Recapping	6	2 (33.3)	26	4 (15.4)	10	1 (10.0)
Other	41	3 (7.3)	27	4 (14.8)	8	2 (25.0)
	**Total**	**with SED**	**Total**	**with SED**	**Total**	**with SED**
Reason ^5^	**n = 315**	**n = 116**	**n = 371**	**n = 103**	**n = 85**	**n = 20**
High workload, time pressure	87	20 (23.0)	56	10 (17.8)	5	2 (40.0)
Distraction by patients or surrounding ^6^	82	22 (26.8)	127	34 (26.8)	24	4 (16.7)
Unexpected patient movement	64	37 (57.8)	79	23 (29.1)	30	10 (33.3)
Problem when using the safety device ^7^	50	31 (62.0)	56	24 (42.9)	9	3 (33.3)
Problem with the safety container ^8^	29	6 (20.7)	50	11 (20.0)	15	1 (6.7)
Inappropriate personal protective equipment	3	0 (0.0)	3	1 (33.3)	2	0 (0.0)

^1^ Composition as described in Table 3. ^2^ Blood collection needle, butterfly needle, venous catheter access. ^3^ Tweezers, dental hygiene instrument, intramuscular needle, lancet. ^4^ Working on sharps container. ^5^ 20 cases were deleted because of missing data. ^6^ Lack of space. ^7^ Safety mechanism could not be triggered mechanically, lack of training in handling of safety device. ^8^ Overfilled sharps container, lack of sharps container near the workplace.

## Data Availability

Aggregated data used in this study are available from the corresponding author on request.

## Abbreviations

The following abbreviations are used in this manuscript:

PSI	Percutaneous sharps injuries
HCW	Healthcare workers
HIV	Human immunodeficiency virus
SED	Safety-engineered devices
TRBA	Technical rule for biological agents
EBN	European Biosafety Network
BGW	German accident insurance provider for non-state institutions within the health and welfare service sectors
NSSI	Needlestick and sharps injury
FTE	Full-time equivalents
DGUV	Umbrella organization of German accident insurance institutions
